# Assessing the Present and Future Habitat Suitability of *Caligus rogercresseyi* (Boxshall and Bravo, 2000) for Salmon Farming in Southern Chile

**DOI:** 10.3389/fvets.2020.615039

**Published:** 2021-02-09

**Authors:** Manuel Lepe-Lopez, Joaquín Escobar-Dodero, Natalia Zimin-Veselkoff, Claudio Azat, Fernando O. Mardones

**Affiliations:** ^1^PhD Program in Conservation Medicine, Facultad de Ciencias de la Vida, Universidad Andres Bello, Santiago, Chile; ^2^Facultad de Ciencias de la Vida, Centro de Investigación para la Sustentabilidad, Universidad Andres Bello, Santiago, Chile; ^3^Department of Veterinary Population Medicine, University of Minnesota, St. Paul, MN, United States; ^4^School of Veterinary Medicine, Pontificia Universidad Católica de Chile, Santiago, Chile; ^5^Department of Pediatric Infectious Diseases and Immunology, School of Medicine, Pontificia Universidad Católica de Chile, Santiago, Chile

**Keywords:** sea lice, copepod, ecological modeling, habitat suitability model, maximum entropy, climate change, aquaculture, salmon farming

## Abstract

The sea louse (*Caligus rogercresseyi*) is the most relevant parasite for the farmed salmon industry in Chile, the second largest producer worldwide. Although spatial patterns of *C. rogercresseyi* have been addressed from data obtained from established monitoring and surveillance programs, studies on its spatial ecology are limited. A wide geographic distribution of *C. rogercresseyi* is presumed in Chile; however, how this species could potentially be distributed in space is unknown. Our study presents an analysis of the habitat suitability for *C. rogercresseyi* in the entire area occupied by marine sites of salmon farms in Chile. Habitat suitability modeling was used to explore the likelihood of species spatial occurrence based on environmental characteristics. Due to the expanding salmon industry in southern Chile, we studied *C. rogercresseyi* habitat suitability models for present (average of 2005–2010) and two future projections (2050 and 2100) under different climate change scenarios. Models were constructed with the maxent algorithm using a large database of spatial *C. rogercresseyi* occurrences from the Chilean fisheries health authority and included 23 environmental variables obtained from the Ocean Rasters for Analysis of Climate and Environment (Bio-ORACLE). Habitat suitability models indicated that water temperature, water salinity, and current velocity of waters were the most important characteristics limiting *C. rogercresseyi* distribution in southern Chile. Habitat suitability models for current climate indicated a heterogeneous pattern with *C. rogercresseyi* being present in waters with temperature range 12.12–7.08°C (sd = 0.65), salinity range 33.7–25.5 pss (sd = 1.73), and current water velocity range 0.23–0.01 m^−1^ (sd = 0.02). Predictions for future projections in year 2050 and year 2100 suggest new clumped dispersion of the environmental conditions for *C. rogercresseyi* establishment. Our results suggest complexity and a wide dispersion of the biogeographic distribution of the *C. rogercresseyi* habitat suitability with potential implications for control strategies and environmental issues for salmon farming in Chile. Further investigations are required into *C. rogercresseyi* distribution in southern Chile considering the possible effect of climate change.

## Introduction

The sea louse (*Caligus rogercresseyi*) is the most relevant parasite of salmon farming in Chile, the second-largest salmon producer worldwide. This marine copepod feeds on the mucus, skin, and blood of parasitized fish, altering the osmotic barrier, reducing appetite, decreasing food conversion, damaging the carcass, and as a consequence producing substantial economic losses (1). The three species of salmonids intensively farmed in Chile: Atlantic salmon (*Salmon salar*), rainbow trout (*Oncorhynchus mykiss*), and coho salmon (*O. kisutch*), are all susceptible to *C. rogercresseyi* infection (2). The life cycle of this copepod species includes eight different development stages: two nauplius and one copepodid, four chalimus, and the adult (3). The first three stages are planktonic, and the others are parasitic. The cost of the salmon production unit in Chile increases USD$1.4/kg for the treatment against *C. rogercresseyi* (4). In addition, the economic impact of sea lice globally is estimated at USD$650 million per year, considering the loss in weight gain and the predisposition to secondary diseases (5).

A wide geographic distribution of *C. rogercresseyi* is presumed according to the reports of salmon farming in southern Chile. Since the beginning of salmon farming in the 1980s on region X [Los Lagos], sea lice infestation has been reported with farms producing rainbow trout (*O. mykiss*) particularly on the island of Chiloé (2). With the advance of the salmon industry in the 1990s toward the region XI [Aysén], sea lice infestations were also confirmed in farmed Atlantic salmon (*S. salar*). These two regions are considered as northern Patagonian fjords, a complex and heterogeneous topographic and oceanographic habitat composed of fjords, gulfs, channels, and semi-enclosed water bodies (6). However, for the decade of 2000s, a parasitosis prevalence of 53.4% was estimated in salmon farms in both regions with a decreasing gradient for the farms located further south (7). Besides, with the health crisis due to Infectious Salmon Anemia virus (ISAV) (8), some farming companies expanded their salmon production to more isolated, pristine, and cold sites in the southernmost part of the country. Recently in 2017, the presence of *C. rogercresseyi* was reported for the first time in region XII [Magallanes], the southernmost sites with salmon farming in Chile, even though these isolated farms represent <10% of the industry (5).

Usually, the distribution patterns of *C. rogercresseyi* have been approached with an epidemiological focus or considering the effectiveness of control measures interests (7, 9–15), yet studies of spatial ecological patterns of this parasite are rare (16). However, how this species is distributed in space is unknown in Chile (17). Furthermore, this ambitious question can be partially answered by addressing *C. rogercresseyi* presence as a function of environmental conditions in the area occupied by the salmon industry. Predicting the spatial distribution of the environmental conditions relevant to *C. rogercresseyi* is necessary to know the potential geographic range where the health and economic impacts of this parasite to salmon farming in Chile are expected.

According to the ecological niche theory, a species only thrives within definite ranges of environmental conditions (18, 19). The habitat suitability of a species is the predictions of presence/absence of the likelihood of occurrence based on environmental characteristics in a statistical modeling process (20). However, the concept of suitable habitat models (HSMs) should be differentiated from ecological niche modeling, considering that the latter in a function that links the fitness of the species to their environment (21). Therefore, ecological niche modeling is a quantitative framework that includes evolutionary processes, interspecific relationships, migration (movement), and other biotic and abiotic aspects for individuals (22). The HSMs framework estimates a qualitative output (usually the presence) considering the relationship between environmental variables of biological relevance for the individuals.

This paper presents an analysis of the habitat suitability for *C. rogercresseyi* in southern Chile. The ongoing expansion of the salmon industry in Chile is an important characteristic of this parasitic dynamic. Although salmon production fell from 700,000 to 500,000 tons due to the ISAV health crisis in 2008, the industry has shown a growing recovery to 900,000 tons in 2014 (23). A central issue is a transnational and capitalistic salmon industry supported by the idea of economic growth from the national state. The foregoing poses health and environmental challenges for the development of this activity in the future. For this reason, we studied the predictions for HSMs using a presence-only method (Maxent) for the present (average of 2005–2010) and future projections (2050 and 2100) under different climate change scenarios. The importance of our mapping of HSMs is to know the spatial heterogeneity that *C. rogercresseyi* could exhibit under different scenarios and predict the possible impacts of this important health issue to the salmon industry.

## Methods

### Study Area

**Figure 1:**
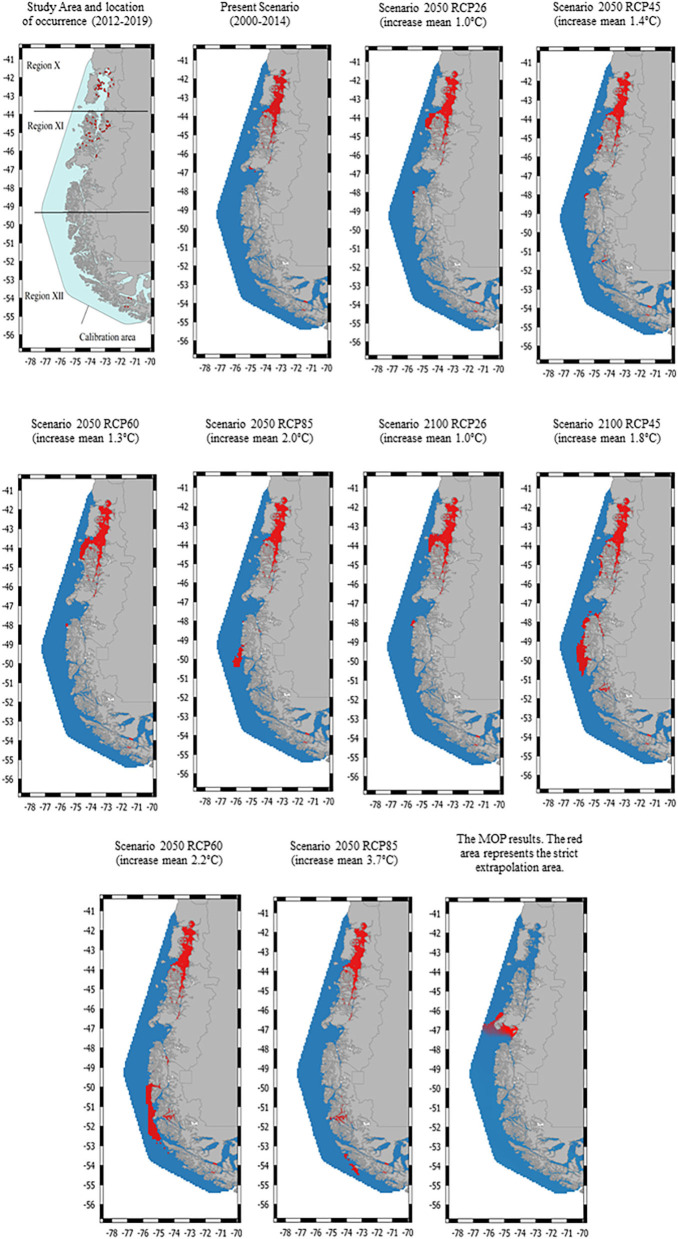
The study area consists of three geographical regions in Chile: region X [Los Lagos], region XI [Aysén], and region XII [Magallanes]. The locations of the *Caligus rogercresseyi* spatial occurrence used for habitat suitability models are indicated by red dots. Habitat suitability maps for *C. rogercresseyi* (red areas) in southern Chile at present and two future projections (2,050 and 2,100) under different climate change scenarios using a maximum entropy modeling (Maxent). Different representative concentration pathways (RCP 2.6–8.5) represent different greenhouse gases concentrations.

The study area covers a latitudinal extension of ~1,500 km of coastline waters in southern Chile (41 to 54°S), across three geographical regions (region X [Los Lagos], region XI [Aysén], and region XII [Magallanes]) where salmon production is established (Figure 1). This area covers 100% of the country's salmon farming activity. The average annual water temperature is 9.1°C (minimum of 6.6, maximum of 12.3), and the average annual water salinity is 33.32 pps (minimum of 25.38, maximum of 34.01).

### *Caligus rogercresseyi* Data

The estimation of HSMs is based on a large database of spatial occurrences of *C. rogercresseyi*. We obtained the data from the monitoring program for *C. rogercresseyi* [Specific Sanitary Program for the Surveillance and Control of Caligidosis—SSPSC] maintained by The Chilean National Fisheries and Aquaculture Service (Sernapesca) (24). The objective of the SSPSC program is to monitor *C. rogercresseyi* abundance in all active salmon farms throughout time and assess sanitary measures to control this parasitic infestation. The trained staff at each farm takes a random sample of 40 fish for the count total of individuals by parasitic phases of three categories: juveniles phases (includes chalimus I, II, III, and IV), mobile adults (males and no gravid females), and gravid female (egg sacs strings) (25). The data obtained of SSPSC consists of *C. rogercresseyi* abundance and the geographic location (decimal latitude and longitude coordinates) of all farms in operation from January 2012 to December 2018 on region X, XI, and XII in southern Chile. We consider *C. rogercresseyi* occurrences in each report of the presence of ovigerous female in the study area. The rationale behind this is that the gravid female indicates that the life cycle of the copepods is complete in a geographical site as a biological occurrence. Besides, the gravid female phase can be identified with the naked eye in the field as chains of eggs are visible and it is ~5 mm long (3). A *C. rogercresseyi* occurrence database was generated in MS Excel, including geographic coordinates. Duplicate occurrence records and absolute zeros of farm abundance during uptime were removed since it is assumed that the susceptible fish host is present but there is no evidence of interaction with the parasite and the design of the SSPSC program is to estimate the infection (the presumed false absences are very uncertain) (26). In addition, duplicate occurrences were removed because it is advisable to avoid multiple records for the same species in the same grid cell for the modeling process as salmon farms can report the *C. rogercresseyi* more than once. A parasite prevalence was estimated without distinction between old and new farms infected (number of positive farms by study period/number of active farms by study period). To determine optimal prevalence thresholds for inclusion as an occurrence in the modeling dataset, we created subsets based on the 100, 80, 60, 50, and 20 percentile prevalence distribution. In this case, the original data included records from 790 active farms for the 100 percentile (corresponding active farms by prevalence thresholds 80, 60, 50, and 20 percentile were 632, 474, 395, and 158 farms).

### Accessible Location

The HSMs require a geographic location that is accessible to the species for a certain period (20). Thus, we defined a boundary area given the dispersal abilities of *C. rogercresseyi* based on occurrences in southern Chile. According to the available knowledge, the planktonic and infective phases can be dispersed 30 km around (27). Therefore, we imported the MS Excel occurrence database into a Geographic Information System (Quantum GIS, version 3.4) to estimate a buffer area. A layer polygon in shapefile was generated covering an area around 30 km from each occurrence of the database as an accessible location for *C. rogercresseyi* in the area occupied by the salmon industry.

### Environmental Variables

Another important assumption of the HSMs is the identification of the environmental conditions necessary for the subsistence of the species. We used 23 environmental variables of the Bio-ORACLE (ocean rasters for analysis of climate and environment) marine surface dataset (http://www.bio-oracle.org) provided in grids with cells equally spaced in latitude and longitude with a spatial resolution of 5 arcmins (c. 9.2 km) (28). Bio-ORACLE is a global dataset consisting of geophysical, biotic, and climate variables for a present (average 2005–2010) and two future projections (2050 and 2100) based on Representative Concentration Pathways (RCP). The RCP projections describe different climate futures scenarios considered possible depending on the amount of greenhouse gases emitted in the years to come at the following temperature thresholds: RCP 2.6 (global temperature rise <1°C by 2100), RCP 4.5 (>1°C), RCP 6.0 (>2°C), and RCP 8.5 (>3°C). In other words, RCP 8.5 represents the worst-case climate change scenario (29). The Bio-ORACLE raster layers were imported into QGIS for clipping the “accessible location” proposed for the *C. rogercresseyi* using the shapefile as a mask.

To reduce the dimensionality of the 23 environmental variables, we examined with a Principal Component Analysis (PCA) using the PCA4cd plugin (QGIS, version 3.4). This analysis summarizes uncorrelated variables giving significant environmental information for HSMs (20). After reducing the number of variables by a statistical test, biological criteria were applied to the *C. rogercresseyi* life cycle, limiting the number of variables to conditions of water temperature °C, water salinity pss (practical salinity scale), and currents water velocity m^−1^ (3, 16, 30–32).

### The Modeling

The HSMs analysis is limited to occurrence data (presence) because the SSPCS program has a sanitary approach to establish farms that need chemical control for the parasitic infestation. Therefore, there are no directed efforts to estimate the strict absence of *C. rogercresseyi*. We use Maxent (maximum entropy modeling) as an algorithm that simulates pseudo-absences to complement the dependent variable (33). In this case, Maxent works as a Generalized Linear Model with Poisson distribution where the number of occurrence counts is a function of environmental variables representing a prediction of indices as habitat suitability for *C. rogercresseyi* on the study area (34). However, like any generalized prediction of environmental conditions, these models cannot capture ecological interactions, limiting themselves to an estimate where the species is most likely to occur. The foregoing should be considered with caution because these estimates do not represent quantitative estimates that suggest a greater or lesser occurrence of the species in the study area.

To fit HSMs Maxent models, we used specific settings: raw output (this feature estimates the ratio of the relative suitability of one pixel vs. another's pixel within the accessible location), 20% of random test (a quantity of the data is withheld every time and used for testing), with 100 replicates (amount of k-folds models to train), bootstrap (training data is selected by sampling with replacement from the presence points), and deactivating extrapolate and do clamping options (no projections outside the limits of the training data). Then, we made a range of models for the present using different regularization multiplier to avoid overfitting (0.5, 1.0, 1.5, 2.0, 2.5, 3.0, 3.5, 4.0, 4.5, 5.0). The output directory for these models was the environmental variables of the present (2005–2010). For these models, we estimated the Akaike information criterion (AIC) using the toolbox of Niche Analyst software (Niche A, version 3.0) (35). According to the reference framework of Multimodel Inference, under similar conditions, the models with the lowest AIC presents better-estimated values for a suitable habitat (36). To fit HDM Maxent models for two future projections (2050 and 2100), we used the same specific settings applying them to four climate change scenarios (RCP 2.6, RCP 4.5, RCP 6.0, and RCP 8.5), and AIC values were estimated for the range of the different regularization multiplier (0.05–5.0) selecting the AIC smallest model. The modeling process was developed using Maxent 3.4.1 (33).

To evaluate the models based on each prevalence threshold, we use the Area Under the Curve (AUC) by summing the estimates values for the Receiver Operating Characteristic (ROC). The ROC is a graphical estimate of sensitivity against 1-specificity for all possible thresholds of the model. The summary statistic of AUC calculates values between 0 and 1 from ROC, where values >0.5 suggest that our model is better than random (37). In this case, the AUC values >0.5 and near 1 are useful to compare the predictive strength of multiple only presence data models using the same data (38). We selected as the best model the prevalence threshold for which models showed the highest AUC value. To evaluate HSM uncertainty, we detected areas of strict extrapolation as places where environmental conditions are non-analogous to those in areas across which the models were calibrated (39). The mobility-oriented parity metric (MOP nearest 5% of reference cloud) was used to identify such extrapolative areas resulting from calibration areas, compared with conditions under present and future climate scenarios using “ntbox” R package (40). Then, we excluded areas with strict extrapolation to represent only potential suitable areas with higher levels of certainty. After establishing the best model by AUC close to 1, we used the mean of the 100 replicates to generate a binary map (QGIS version 2.18) using as threshold the highest value that included 95% of occurrences (omission error 5%).

## Results

**Figure 2:**
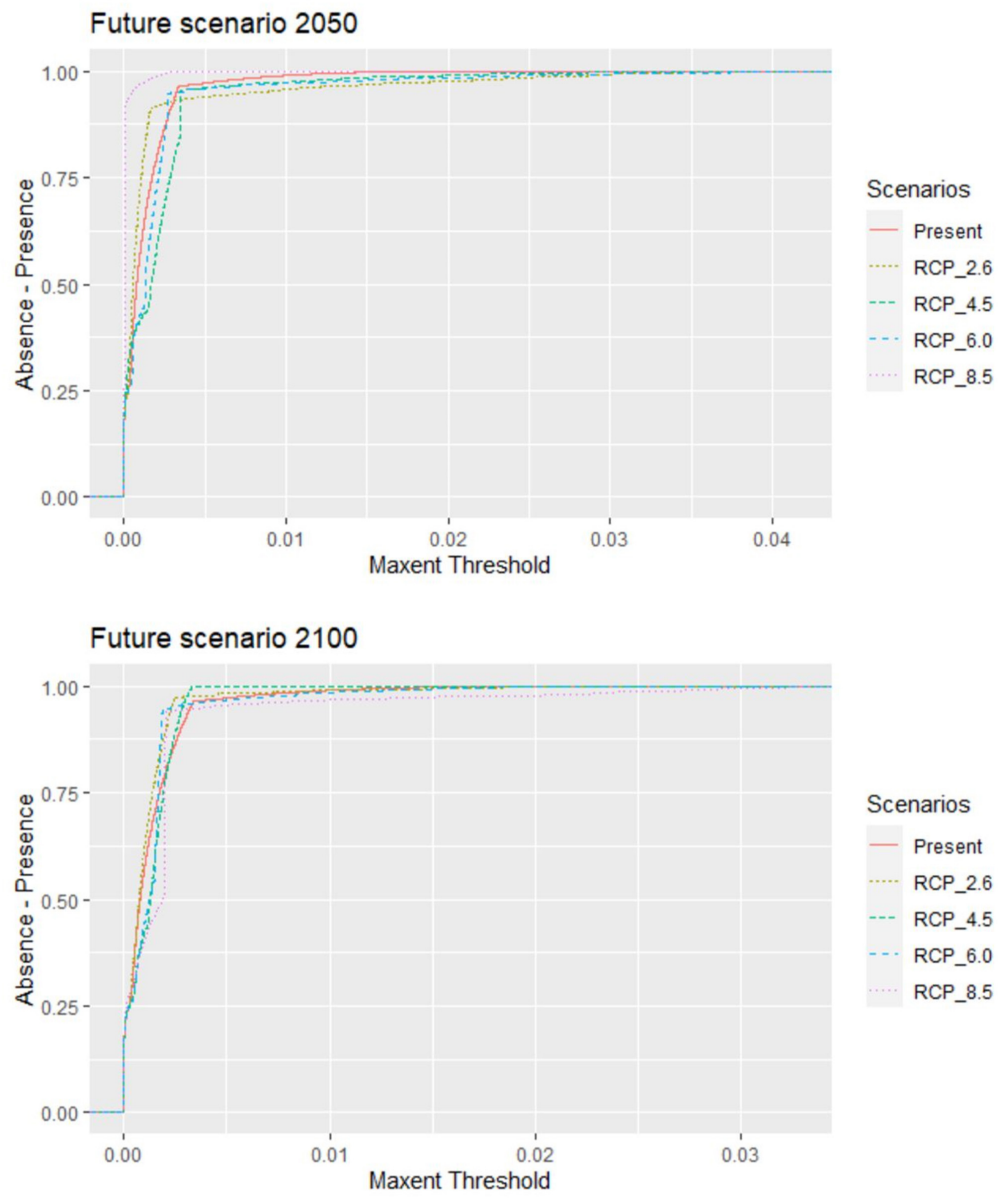
*Caligus rogercresseyi* habitat suitability projections for the area occupied by salmon farms in southern Chile. The behavior for the Maxent threshold (dashed line) of different Representative Concentration Pathways (RCP 2.6, 4.5, 6.0, and 8.5) in two different projection future scenarios (2,050 and 2,100) compared to the present scenario 2000–2014 threshold (continuous line). The suitable conditions predicted for future scenarios (threshold) are similar to the present conditions, but they differ because different feature types allow different possible shapes of response curves.

The original data included 68,114 records from 790 active farms during the study period with a 92% parasitic prevalence. A total of 728 *C. rogercresseyi* occurrences from salmon farms were used to select the HSMs (Supplementary Material). Of these occurrences, 308 were established in Region X [Los Lagos], 409 in Region XI [Aysén], and 11 in Region XII [Magallanes] during the years 2012–2018. According to PCA analyses, only three environmental variables are necessary to explore the variability of the environmental conditions in the study area: mean water temperature, mean water salinity, and mean current water velocity. In addition, as low temperatures limit the development of *C. rogercresseyi* according to the biological cycle, we added a temperature minimum to the environmental variables set (this characteristic present correlation <0.7). The most parsimonious HSMs based on AIC scores included a regularization multiplier value of 0.5 in Maxent software (default value is 1). Besides, Maxent models performed better than random for each time prediction and showed a good fit (AUC > 0.95). Predicted habitat suitability for *C. rogercresseyi* using maximum entropy models is shown in Figure 1. The Maxent thresholds for climate change scenarios are shows in Figure 2.

The majority of predicted suitable habitat areas were near the coastline of the study area, where most salmon farms are located. However, a heterogeneous pattern is observed with a greater *C. rogercresseyi* presence in the northern sites. Therefore, the suitable habitat for *C. rogercresseyi* at current climate is widely distributed in waters with temperature range 12.12–7.08°C (sd = 0.65), salinity range 33.7–25.5 pss (sd = 1.73), and current water velocity range 0.23–0.01 m^−1^ (sd = 0.02) across the study area. Besides, in region XII a clumped area (54° S, 71° W) is observed with suitable environmental conditions for *C. rogercresseyi*. In addition, the suitable habitat for *C. rogercresseyi* is limited by water temperature minimum <5.5°C across the study area. Future predictions for 2050, under RCP 2.6 estimate a slight advance of the suitable habitat near Guamblin Island National Park (44° S, 74° W) toward the south in region XI but without uniform distribution toward region XII (Figure 1). Future predictions for 2050 under RCP 4.5 estimate a loss of the suitable habitat (44° S, 74° W) and a slight advance of new areas in River Island (45° S, 74° W) toward the south in region XI but without uniform distribution toward region XII (Figure 1). In the case of the 2050 predictions under RCP 6.0 and 85, a loss of the RCP 2.6 and RCP 4.5 new areas is perceived in region XI but with an increase in suitable areas in region XII on Mornington Island (49° S, 75° W), in sites not occupied by salmon farms under current conditions. Future predictions for 2100 predictions suggest greater coverage areas compared to models for 2050, mainly in Region XII. Model predictions for 2100 under RCP 26 indicated a focus in Region XI in the north part of Campana Island (48° S, 75° W). In addition, under RCP 45 projections the new area extends continuously to Region XII from Merino Jarpa Island (47° S, 74° W) to the northern part of Duke of York Island (50° S, 75° W) and an isolated area on Rennell Island (51° S, 74° W). Future predictions 2100 under RCP 60 indicated the contraction of the suitable habitat observed in the RCP45 projections and a new continuous area in region XII from the Bernardo O‘Higgins national park (50° S, 74° W) to the south of the Alacalufes Natural Reserve (52° S, 73° W). Future predictions for 2100 under RCP 85 suggest a partial reduction of the areas, maintaining favorable conditions in Puerto Natales (47° S, 74° W) and new areas appearing to the south between the Recalada island (53° S, 74° W) and the Carlos island (54° S, 73° W) in region XII. The MOP results showed areas of strict extrapolation as uncertainty predictions when looking at results for calibration areas agreement among them, mainly in Region XI (46° S, 74° W) near Laguna San Rafael National Park, and in Region XII (52° S, 69° W) near San Gregorio Village (Figure 1).

## Discussion

We used presence-only maximum entropy modeling to represent the habitat suitability of *C. rogercresseyi* in southern Chile at present and projected for 2050 and 2100. Mapping outputs of HSMs suggest that habitat suitability almost entirely covers the area occupied by the salmon industry overpass Los Lagos to Aysén regions. Besides, future predictions for the years 2050 and 2100 suggest growing suitable habitat contiguous to the Aysén region with an isolated and delimited new area of favorable environmental conditions for *C. rogercresseyi* in the Magallanes region. We cautiously suggest avoiding farming use of these newly suitable areas shown in future predictions (Figure 1). The parasite dynamics would be similar in these new areas to that observed in northern areas, which would be added to the costs of maintaining salmon production and control measures in more remote sites (41). This heterogeneous pattern and the environmental variables detected involved have potential implications in *C. rogercresseyi* biogeography, environmental issues, and control strategies.

Using the maxent algorithm to project probable future scenarios based on current data entails uncertainty. Other statistical methods as generalized linear models or generalized additive models consider strict species absence reducing uncertainty (42). A fundamental limitation of presence-only data using maxent is that some sites in the study area are sampled more intensively than others (38). For our data, it is relevant to consider that *C. rogercresseyi* infection requires a susceptible host density at existing farms (43, 44). Therefore, we obtained models that combine the *C. rogercresseyi* habitat suitability distribution with the distribution of salmon farms. On the other hand, MOP results identify high uncertainty areas with strict extrapolation risks as a key approach in this regard, suggesting prediction variation depending on the environmental data used to create models.

Temperature is the most important environmental characteristic to predict the *C. rogercresseyi* habitat suitability, with less cold waters within the study area favoring its occurrence. Development rates of parasitic copepods are dependent on temperature, altering their geographic distribution and increasing infestations in farms at higher temperatures (45). Besides, it is known that the development time of the different phases of *C. rogercresseyi* is affected by temperature (3). The complete life cycle can take 45 days at a mean temperature of 10.3°C. However, the life cycle can be completed in just 18 days at a temperature of 16.7°C. This is in agreement with our results in which we found more suitable habitats for *C. rogercresseyi* in Los Lagos and Aysén regions due to warmer waters compared to Magallanes region. On the other hand, predictions based on future climate change scenarios suggest that habitat suitability can be expanded in a specific area of Magallanes region. Moreover, this should be taken with caution because increasing the mean temperature could potentially reduce the development time of the parasitic phases or could also cause distress in the copepod dynamic (e.g., patterns prediction for 2100 in scenarios RCP 4.5, 6.0, and 8.5; Figure 1) (46, 47).

Minimum temperature defines the environmental limit of the geographic range for *C. rogercresseyi* in our models. The temperature threshold of <4.2°C not only stops the development of all phases but also causes the death of this species of copepod (3). This threshold is only observed for the southernmost sites in our study area where there are no occurrences of the species. However, the increase in the mean temperature expected with climate change projections would modify the minimum temperature threshold, expanding the habitat suitability for *C. rogercresseyi* in Chile (29). This implies the need to understand how the salmon farming in Chile interacts with the environment, mainly the conflicts over marine space with other aquaculture activities and whether a more sustainable industry is possible (23).

Salinity and current velocity are the variables with the least importance in our models. In other species of sea lice in the northern hemisphere (*Lepeophtheirus salmonis* and *C. elongatus*), low salinities <30 pss have been observed to decline reproductive and survival rates (48). For the species *C. rogercresseyi*, a variable tolerance and sensitivity to salinities <20 pss have been described (31). Experimental studies show variations between 0, 15–100, 45–100, and 100% *C. rogercresseyi* survival rate at salinity concentrations of 0, 10, 20, and 30 pss, respectively. However, the slight salinity effect observed in our models may be because the low concentration is associated with specific geographical sites, such as estuaries and brackish waters. Concerning velocity current, it is understood that current can flush the planktonic phases and the infective phase toward neighboring farms (27). Nonetheless, this effect could be limited on a large scale, since it has been reported that for *L. salmonis* the current can transport the phases only between around 7.3 and 10 km (49). In addition, information is still lacking on the role of current velocity in the transport of planktonic phases of *C. rogercresseyi* in the complex fjord systems of southern Chile.

The pattern predicted by our models is consistent with previous reports of this species of copepod in Chile. The first spatial and temporal description of *C. rogercresseyi* proposes a cluster pattern according to the number of farms in operation during 1999–2000 (50). Subsequent analyses with data from 2007 identified a decreasing pattern from north to south on the abundance of *C. rogercresseyi* in salmon farms (7, 51). However, our study shows a clumped advance of the suitability conditions under the assumptions of climate change, aggravating sea lice infestations, and their economic and environmental consequences in the medium-term future for Chile. Besides, according to our results, a latitudinal gradient is observed in the suitable habitat of *C. rogercresseyi*, where its likelihood of occurrence increases toward the north in our study area. It is necessary to expand the biogeographic knowledge of the *C. rogercresseyi* due to the trend of expansion of aquaculture in the face of the reduction of the global capture fisheries. Besides, there are hypotheses about a wider distribution range of the *C. rogercresseyi* from Peru to Argentina (passing through Chile), complicating the control strategies by the natural sources of the parasite (17, 52).

The habitat suitability distribution proposed by our models reflects a challenge for sea lice control strategies in Chile. The environmental conditions of less cold temperature >10°C, salinities > 25 pss, and low velocity of currents will guarantee the thrive of the *C. rogercresseyi* in Los Lagos and Aysén regions for the next 80 years. Consequently, the loss of sensitivity of sea lice to chemical control can be repeated in future scenarios in southern Chile. Before 2007, emamectin benzoate was exclusively used to control *C. rogercresseyi* infestations, causing loss of sensitivity to this antiparasitic drug (53). After 2007, the health authority approved the use of deltamethrin, followed by diflubenzuron in 2009, cypermethrin in 2010, and azamethiphos in 2013 (14). The implementation of azamethiphos as a new alternative of chemical control in 2013 coincides with an increase of *C. rogercresseyi* abundance at salmon farms in region X [Los Lagos] (54). Although up to now the use of azamethiphos maintains limited parasitic loads, it is necessary to improve the proper management of chemicals and propose non-chemical options due to the expansion and emergence of antiparasitic-resistant *C. rogercresseyi*.

In addition, the impacts of *C. rogercresseyi* chemical control on non-target aquatic organisms is an environmental concern of relevance under the projections of geographic expansion for this copepod in future scenarios. Emamectin benzoate, diflubenzuron, deltamethrin, cypermethrin, and hydrogen peroxide at the recommended dose reduce the feeding and mobility, producing paralysis and dead in copepods and causing >95% mortality in another zooplankton and phytoplankton organisms under laboratory conditions (55, 56). Otherwise, cypermethrin and deltamethrin in high doses cause 100% mortality in the marine amphipods *Monocorophium insidiosum* (57). Furthermore, the synergistic effect of cypermethrin and deltamethrin combination on crustacean *Daphnia magna* has been described to cause superior toxicity to the half-maximum effective concentration [EC50; (58)]. In larger crustaceans, specifically the stage IV lobsters (*Homarus americanus*), a high concentration of azamethiphos produces agitation, relaxing erratically, and aggressiveness and causes 40% mortality in 4 h of experimental continuous exposure (59). Emamectin benzoate lacks these toxic effects in *H. americanus* (60). However, it is alarming that the pelagic marine ecosystem of Chile is characterized by high endemism of copepods (61–63), marine organisms very sensitive to toxicity effects of chemical control used against *C. rogercresseyi*.

Other impacts of *C. rogercresseyi* include their interaction with native fish, where low abundances are observed for native fish Rock cod (*Eleginnops maclovinus*) and Chilean silverside (*Odontesthes regia*) (2). However, *C. rogercresseyi* parasitism on salmonid production species shows high abundances and infestations in Atlantic salmon (*S. salar*) and rainbow trout (*Oncorhynchus mykiss*), while low abundances are shown in coho salmon (*O. kisutch*) due to a better immune response mediated by macrophages (64). Besides, stocking density (>22 kg m^−3^), the season of the year (autumn), depth of water at the farm and cage location (>50 m), and chemical control (>1 week later) are all factors associated with higher *C. rogercresseyi* infestation rates (16, 51). Furthermore, the growing trend of salmon farming worldwide expects a growing host density, causing negative feedback for this host–parasitic dynamics (44).

The maps from HSMs provide a useful guide to hypothesize the occurrence of *C. rogercresseyi* in different climate change scenarios, mainly for the Magallanes region. However, these models are limited by only-presence data of salmon farms in Chile. We lack interpretation to establish the limited dispersal and potential distribution for using pseudo-absences. In addition, our study area could be small for the spatial distribution of this species by ignoring biotic interactions (competition, commensalism, multi-species studies) and the migration process (20). In this respect, we limit our predictions to the area of calibration of the models, with utility only for the area occupied by the salmon industry in Chile. Interpolation is acceptable and necessary because we used the largest database of *C. rogercresseyi* occurrence that is available. Our results suggest complexity and a wide dispersion of the biogeographic behavior of the *C. rogercresseyi* in the south of Chile; further investigations are required in these areas.

## Data Availability Statement

The original contributions presented in the study are included in the article/Supplementary Material, further inquiries can be directed to the corresponding author.

## Author Contributions

ML-L wrote the manuscript and analyzed and interpreted the data. JE-D and NZ-V collected the data and prepared the figures. CA review and wrote the manuscript. FM wrote the manuscript and conceived and planned the study. All authors contributed to the article and approved the submitted version.

## Conflict of Interest

The authors declare that the research was conducted in the absence of any commercial or financial relationships that could be construed as a potential conflict of interest.
